# Understanding voluntary human movement variability through data-driven segmentation and clustering

**DOI:** 10.3389/fnhum.2023.1278653

**Published:** 2023-11-28

**Authors:** Jean-Francois Daneault, Brandon Oubre, Jose Garcia Vivas Miranda, Sunghoon Ivan Lee

**Affiliations:** ^1^Department of Rehabilitation and Movement Sciences, Rutgers University, Newark, NJ, United States; ^2^College of Information and Computer Sciences, University of Massachusetts Amherst, Amherst, MA, United States; ^3^Biosystems Lab, Physics Institute of Universidade Federal da Bahia, Salvador, Brazil

**Keywords:** motor control, action control, movement control, movement kinematics, movement elements, upper limb

## Abstract

Recently, we proposed a novel approach where movements are decomposed into sub-segments, termed movement elements. This approach, to date, provides a robust construct of how the brain may generate simple as well as complex movements. Here, we address the issue of motor variability during voluntary movements by applying an unsupervised clustering algorithm to group movement elements according to their morphological characteristics. We observed that most movement elements closely match the theoretical bell-shaped velocity profile expected from goal-directed movements. However, for those movement elements that deviate from this theoretical shape, a small number of defined patterns in their shape can be identified. Furthermore, we observed that the axis of the body from which the movement elements are extracted (i.e., medio-lateral, antero-posterior, and vertical) affect the proportion of the movement elements matching the theoretical model. These results provide novel insight into how the nervous system controls voluntary movements and may use variability in movement element properties to explore the environment.

## 1 Introduction

We recently proposed the concept of movement elements (MEs) (Miranda et al., 2018). This concept is based on the principle that any voluntary movement can be decomposed into one-dimensional point-to-point movements within a Cartesian coordinate system originating at the center of mass. These MEs are related to the minimum-jerk principle and account for the cost of time as proposed by Hoff (1994) and are an extension of the work of Meyer et al. (1982, 1988). While significant additional work is necessary to better understand the importance of MEs in the control of movement, we recently showed that features of MEs are associated with a variety of movements and motor impairments (Miranda et al., 2018; de Lemos Fonseca et al., 2020; Oubre et al., 2020, 2021; Lee et al., 2022; Liu et al., 2022).

Taken together, our prior work highlights that the features extracted from the ME decomposition approach yield important information about the motor system in health and disease. While this sub-movement approach to the evaluation of movement kinematics has been abandoned by most researchers, our prior work clearly indicates the relevance of this approach in characterizing voluntary and involuntary movements, and that the sub-movement approach to the assessment of movement kinematics needs to be revisited as it may have been too hastily dismissed. Here, we investigate the distribution of the variability around the cost function that we have observed in our prior work. We hypothesized that during complex movements, a large proportion of MEs would closely fit Hoff’s cost function while a small portion would exhibit larger deviations. We asked healthy individuals to perform random 3D arm movements (see Supplementary Figure 1 for example) while tracking the movement patterns of their wrist. We observed that a majority of MEs fit Hoff’s cost function as expected, but the MEs that did not could be clustered into a small number of movement patterns across all subjects. This suggests that while most movements are optimized to minimize jerk and time, the variability in movement kinematics is not random but also follows certain patterns.

## 2 Methods

### 2.1 Subjects

We recruited 15 healthy individuals (24.7 ± 4.2; 11 male). Inclusion criteria were: (i) no known motor or neurological impairments affecting voluntary upper-limb movements, (ii) aged 18 years and older. The protocol was approved by the University of Massachusetts Amherst Institutional Review Board (#2018-4722) and all subjects provided informed consent.

### 2.2 Procedure

Subjects were asked to perform continuous 3D random upper-limb movements for approximately 2.5 min, twice. These movements had to span as much of the space as they could without moving their torso (trunk movement was monitored visually by the experimenter and, as such, minimal movements could have occurred, but these would not have impacted the current results because of their relative amplitude compared to the upper-limb movements). As such, no two trials were the same (see Supplementary Figure 1 for an illustration of two trials from two different subjects). The instructions to the subjects were simply: “without moving your trunk, move your arm randomly until we tell you to stop, similar to movements you would do if you were conducting an orchestra.” The intended effect of the guidance was to have very large movements that would span the majority of the subjects’ workspace. In unpublished pilot testing, not providing such an example led to movements that were performed in a significantly restrained space. However, the analytical approach to extract MEs considers the size and velocity of the movement so, while providing this instruction may have altered the movement space covered by the participants, it did not impact the outcomes. Movement patterns of the wrist of their dominant upper-limb were recorded at 100 Hz using a motion capture system (Qualisys AB).

### 2.3 Data analysis

3D marker trajectories were low-pass filtered at 6 Hz and differentiated to obtain velocity time-series. MEs were then extracted using the previously described technique (Miranda et al., 2018; see Supplementary material for details). In short, the velocity time-series, in each Cartesian axis (antero-posterior, medio-lateral, and rostro-caudal) (see Supplementary Figure 2), was decomposed using zero crossings such that the continuous movement was segmented into MEs of zero initial and terminal velocities. The selection of the coordinate system was originally based on the best fit of the MEs to the theoretical model proposed by Hoff (1994). Since ME decomposition can be sensitive to noise (Miranda et al., 2018), we defined thresholds based on the properties of the motion capture system below which MEs would be discarded. Thresholds for ME displacement (1 mm), and time (80 ms) were implemented based on recordings of a stationary marker. This led to the removal of 9.95% of the initially extracted MEs (11.40% from the *x*-axis, 14.40% from the *y*-axis, and 3.55% from the *z*-axis). This represented 0.52% of the duration of all combined time-series (0.46% of the duration of the *x*-axis time-series, 0.88% of the duration of the *y*-axis time-series, and 0.21% of the duration of the *z*-axis time-series). Each of the remaining ME was then spatially and temporally normalized to enable direct comparison.

**FIGURE 1:**
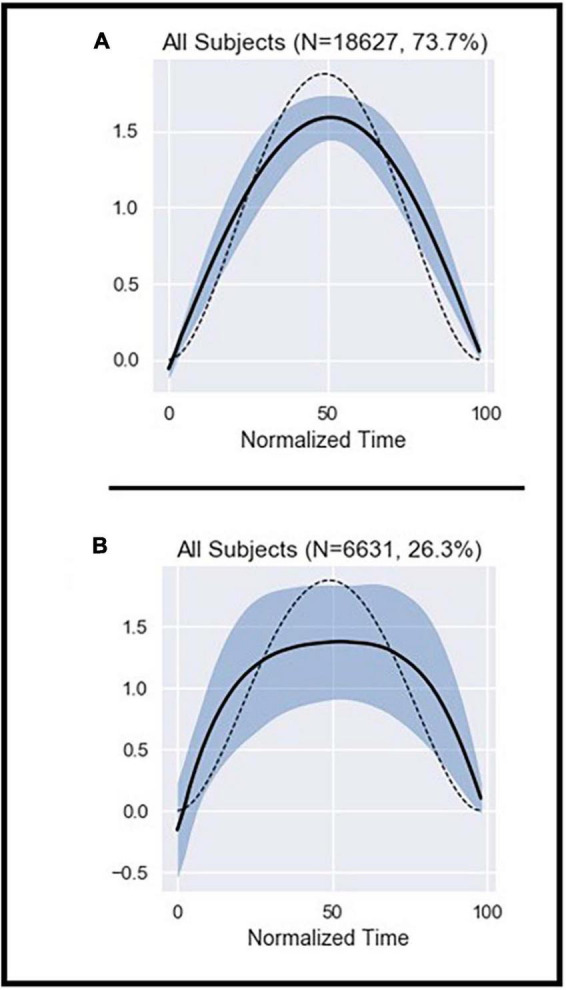
Illustration of the average ME patterns from the homogeneous cluster **(A)** and the outlier cluster **(B)**. The dashed line represents the theoretical shape obtained from Equation 2 (see Supplementary material). The black line represents the average shape of the data included in the graph. The shaded area represents the standard deviation (SD).

Our hypothesis that the majority of MEs would exhibit the bell-shaped velocity profile implies that our dataset should contain a single, dense region of MEs with a similar shape. We identified this region by applying the Density-Based Spatial Clustering of Applications with Noise (DBSCAN) algorithm. Euclidean distance was used as the distance metric between normalized ME vectors because it is a constant multiple of the root mean square error (RMSE). The specific methods used to define the fitness measure developed for this and the parameters used are detailed in the Supplementary material. In short, we defined a fitness measure that constructs a homogeneous cluster containing as many MEs as possible while keeping the variance low (Supplementary Figure 3). Once the dense cluster and the outlier set were identified, we explored whether there were any sub-clusters of movement elements using the *k*-Means algorithm (see Supplementary material; Figure 1). Since the homogeneous cluster is, by definition, one dense cluster, the *k*-Means algorithm, in combination with the Davis-Bouldin Index, was implemented on the outliers set and those parameters were then applied to the homogeneous cluster.

### 2.4 Statistical analyses

Descriptive statistics are in the format *mean* ± *standard deviation*. When comparing the percentage of MEs in the different axes as well as the *K*-value between subjects in the homogeneous cluster and outlier set, normality was assessed using the Kolmogorov-Smirnov test and a one-way analysis of variance (ANOVA) was performed (OriginPro 2018b). Tukey’s *post-hoc* was used to assess significance. One-sample t-tests were performed to assess whether the α values obtained were different from the expected theoretical 2/3. Independent sample *t*-tests were performed to compare mean velocity, duration, and displacement of MEs between the homogeneous cluster and outlier set. The threshold for significance was set to 0.05 *a priori*.

## 3 Results

A total of 24,992 MEs meeting thresholds aimed at minimizing noise were extracted from the motion capture data. DBSCAN was used to identify a single, dense cluster of MEs with homogeneous shape (the “homogeneous cluster”), which accounted for about 74% of all MEs (Figure 1 and Supplementary Figure 4). All other MEs that fell outside of this dense cluster (about 26%) were pooled in the “outliers set” (Figure 1 and Supplementary Figure 4).

The average shape of MEs in the homogeneous cluster closely fits the theoretical cost function whether the subjects were pooled (Figure 1A), or their data examined individually (Supplementary Figure 5). In contrast, MEs in the outliers set showed high variability and deviation from the theoretical cost function (Figure 1B). Note also that the shape of MEs did not significantly differ between the beginning and end of the trials (Supplementary Figure 6).

**FIGURE 2:**
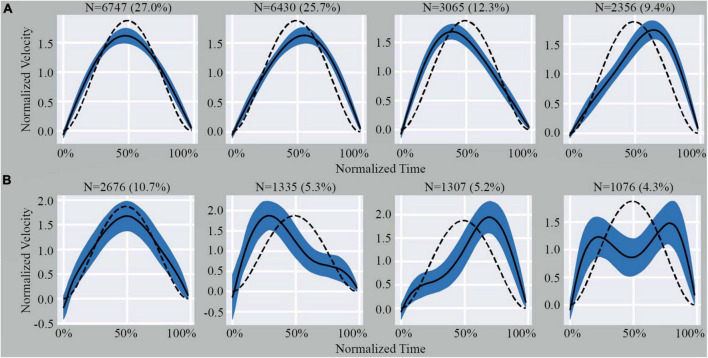
Illustration of the four sub-clusters from the homogenous cluster **(A)** and outlier cluster **(B)**. Indicated above each graph is the number of MEs and percentage of total MEs that fall within that specific pattern. The dashed line represents the theoretical shape obtained from Equation 2 (see Supplementary material). The black line represents the average shape of the data included in the graph. The shaded area represents the SD.

Since the homogeneous cluster was a single highly dense region, it was not possible to identify further sub-clusters using cluster validity indices (Supplementary material). Conversely, the large variability in ME shape observed in the outliers set enabled us to identify an optimal number of sub-clusters using the *k*-Means algorithm and the Davis-Bouldin Index (*k* = 4) (Supplementary Figure 3). Using the same value of *k* to segment the homogeneous cluster and the outliers for comparative analysis, we observed that the shape of MEs within the sub-clusters of the homogeneous cluster showed minimal deviations from the expected model (Figure 2A). However, the sub-clusters of the outliers showed significant deviations from the expected model (Figure 2B).

The percentage of MEs included within the homogeneous cluster differed across subjects (ranging from 57.7 to 87.3%). Furthermore, when we examined the percentage of MEs that were included from each axis on a subject-by-subject basis, there was always a higher proportion of MEs from the *z*-axis within the homogeneous cluster (Table 1). On average, 63% of MEs from the *x*-axis, 65% of MEs from the *y*-axis, and 90% of MEs from the *z*-axis were in the homogeneous cluster. A one-way ANOVA demonstrated a significant difference between the average number of MEs included in the homogeneous cluster from each axis (F: 26.296; df: 2; *p* < 0.05). A Tukey’s *post-hoc* analysis revealed that the average number of MEs included in the homogeneous cluster from the *z*-axis was statistically greater than in the *x*-axis (q: 9.32; *p* < 0.05) and *y*-axis (q: 8.37; *p* < 0.05) while no difference was observed between the *x*- and *y*-axis (q: 0.95; *p* > 0.05). Additional data related to the distribution of MEs based on sub-clusters and axes can be found in the Supplementary material.

**TABLE 1:** Percentage of MEs from each subject that were included in the homogenous cluster per movement axis.

Subj.	X	Y	Z	All axes
1	80.33%	71.70%	99.50%	**84.48%**
2	53.72%	48.60%	82.28%	**60.88%**
3	55.07%	45.12%	83.86%	**61.78%**
4	56.45%	73.45%	88.14%	**74.66%**
5	55.80%	57.69%	79.05%	**64.07%**
6	53.25%	68.60%	96.69%	**75.95%**
7	59.62%	76.05%	82.41%	**73.73%**
8	60.40%	69.68%	96.05%	**77.59%**
9	76.84%	54.90%	87.27%	**71.73%**
10	72.91%	81.17%	100.00%	**86.88%**
11	79.35%	80.67%	98.22%	**87.30%**
12	70.35%	58.49%	98.36%	**76.52%**
13	76.13%	83.47%	89.98%	**83.55%**
14	59.58%	76.44%	93.06%	**78.07%**
15	46.04%	51.73%	74.20%	**57.72%**
All	**64.16%**	**65.70%**	**90.82%**	**74.42%**
All-mean	**63.77%**	**66.52%**	**89.94%**	**74.33%**
All-Std	**11.19%**	**12.69%**	**8.25%**	**9.53%**

For example, Subject 1’s data indicates that 80.33% of the MEs extracted from their *x*-axis, 71.70% of the MEs extracted from their *y*-axis, and 99.50% of the MEs extracted from their z-axis were included in the homogeneous cluster, respectively. Furthermore, it indicates that 84.48% of Subject 1’s MEs were included in the homogeneous cluster. In addition to the subject-level data, The above table provides information about the overall distribution of MEs. For instance, of all the MEs that were extracted in the *x*-axis, 64.16% of them were included in the homogeneous cluster (All). Furthermore, when averaging the subject-level percentages, we can see that on average 63.77 ± 11.19% of the *x*-axis MEs of subjects were included in the homogeneous cluster. The bold values highlight the pooled data results.

**FIGURE 3:**
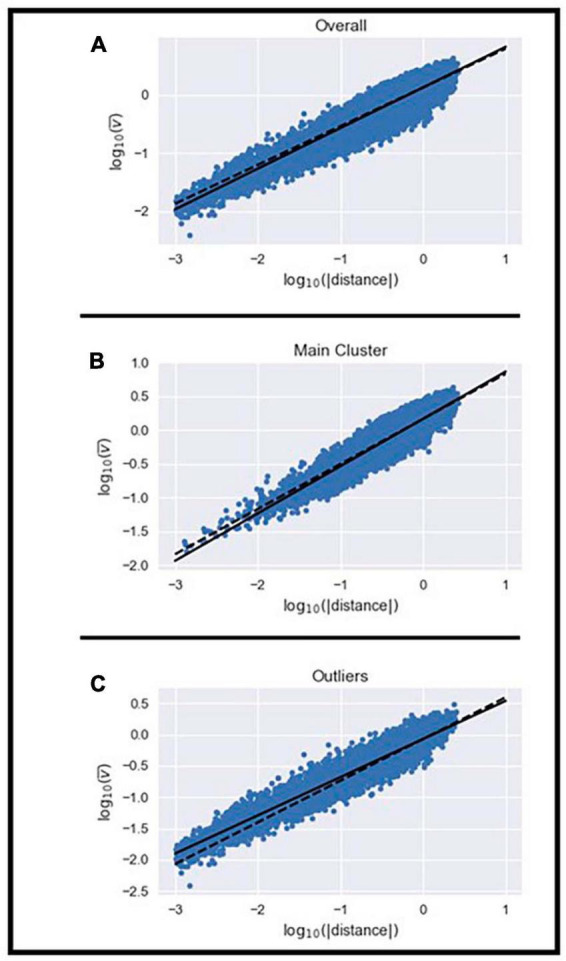
Illustration of the power law relationship between the mean velocity and displacement of all MEs **(A)**, those in the homogenous cluster **(B)**, and the remaining outliers **(C)**.

Next, we examined whether the velocity of the MEs within the homogeneous cluster and the outliers set scaled with their displacement using the 2/3 power law previously described (Miranda et al., 2018). We examined whether the mean velocity, duration, and displacement of MEs were different between the homogeneous cluster and outliers set. Independent *t*-tests showed that mean velocity (*t* = 64.07; df = 24990; *p* < 0.05), duration (*t* = −23.53; df = 24990; *p* < 0.05), and displacement of MEs (*t* = 27.85; df = 24990; *p* < 0.05) were different between the two sets. MEs in the homogeneous cluster had on average, greater mean velocity, shorter duration, and longer displacement than MEs in the outliers set. The scaling exponent (i.e., relationship between mean velocity and displacement) observed for all MEs was 0.709 while the *K*-value from Equation (2) was 6.32 × 10^–4^ ± 61.27 × 10^–4^ (Figure 3A and Supplementary Table 1). The scaling exponent observed for MEs in the homogeneous cluster was 0.703 while their *K*-value was 1.29 × 10^–4^ ± 4.53 × 10^–4^ (Figure 3B and Supplementary Table 1). The scaling exponent observed for MEs in the outliers set was 0.621 while their *K*-value was 4.89 × 10^–3^ ± 33.24 × 10^–3^ (Figure 3C and Supplementary Table 1). These results emphasize that the 2/3 power law relationship between mean velocity and displacement can be observed in the homogeneous cluster and the outliers set even though the average value from the homogeneous cluster deviated slightly from the theoretical 2/3 (t: −3.216; df: 14; *p* < 0.05) and the average value from the outliers set was also statistically different from 2/3 (t: −16.423; df: 14; *p* < 0.05). The range of values in the current study are in line with those reported by Miranda et al. (2018). The larger deviation from the theoretical 2/3 relationship observed in the outliers set was expected because the shape of the MEs in this set differs from the expected model. Furthermore, the *K*-value is approximately 40 times greater in the outliers set than in the homogeneous cluster (t: −2.5934; df: 14.009; *p* < 0.05). We observed significant differences in *K*-values across subjects in the homogeneous cluster (F: 4.257; df: 14; *p* < 0.05) and outliers set (F: 203.72; df: 14; *p* < 0.05). Results show that the *K*-value differs between most subjects in the homogeneous cluster (Supplementary Table 2), however, the *K*-value of the MEs in the outliers set only showed a significant difference in one subject (Supplementary Table 3).

## 4 Discussion

Here, we examined the variability observed around the cost function of the recently proposed ME decomposition approach (Miranda et al., 2018). The ME decomposition approach is an extension of the work done by Meyer et al. (1982, 1988) where sub-movements are extracted based on a Cartesian coordinate system whose origin is located at the center of mass. We have demonstrated that this approach can be successfully implemented to describe a variety of upper-limb movements in healthy individuals (Miranda et al., 2018) as well as to identify short-term features related to motor learning during a balance task (de Lemos Fonseca et al., 2020). We also recently showed that features of MEs are associated with a variety of movements and motor impairments (Miranda et al., 2018; de Lemos Fonseca et al., 2020; Oubre et al., 2020, 2021; Lee et al., 2022; Liu et al., 2022). Therefore, this study aimed to better understand ME characteristics in healthy individuals and explore their potential link with currently accepted motor control concepts.

Our data show that, when healthy individuals perform voluntary random 3D movements of the upper-limb, approximately 74% of MEs match the theoretical shape expected by Equation (1) (see Supplementary material) with no or very slight deviations. The remaining MEs whose shape significantly deviated from the theoretical shape expected by Equation (1) (see Supplementary material) could be further classified into four defined patterns. This replicates and extends our prior work examining MEs of the upper-limb in healthy individuals (Miranda et al., 2018). Prior work has shown that one person at different times, or several different people, never perform a movement in exactly the same way twice (Mathiassen et al., 2002, 2003; Madeleine et al., 2003; Jackson et al., 2009). This is related to the abundancy of the motor system (Latash, 2012) where variance that has no effect on the overall performance across a variety of natural actions helps an abundant system to deal with secondary tasks and unexpected perturbations (Diedrichsen et al., 2010). Our work here extends this concept to MEs during complex upper-limb movements as the observed variability did not lead to any impaired motor performance. Furthermore, it shows that this variability is not random but follows specific patterns in its features and its distribution. Specifically, 63 and 65% of MEs extracted from the medio-lateral and antero-posterior axes, respectively, fell within the homogeneous cluster, while 91% of MEs from the rostro-caudal axis fell within it, highlighting potential differences in motor control strategies when dealing with known forces (i.e., gravity). This is in line with motor adaptation studies where individuals perform tasks within velocity-dependent force fields. These studies have highlighted that a proper balance between stability and flexibility of motor control is necessary so that ingrained skills (e.g., gait, reaching, etc.) remain sensitive to steady and persistent changes in the environment (e.g., gravity) without being disproportionately influenced by incidental events; and where new skills can be learned and developed. In fact, studies have shown that as infants learn to control their limbs, there is a significant impact from gravity during their first few months of life (Kamm et al., 1990; Jensen et al., 2010). This could explain the distribution of MEs within the homogeneous and outliers clusters such that the life-long learned motor patterns performed against gravity are more stable than those not influenced by it. It is important to note that while the MEs within the outliers cluster exhibited variations from the expected theoretical model, our current study cannot determine whether this variability is “good” (having no negative effect on motor performance) or “bad” (leading to detrimental effects on motor performance). As such, future studies should examine this issue. However, as we did not visually observe any “impaired” motor performance, we would hypothesize that the variability in MEs within the outliers cluster is a manifestation of abundance, therefore this could be “good” variability.

Remarkably, the actual shape of the MEs seemed to have little impact on the power law relationship between their mean velocity and displacement previously described (Miranda et al., 2018). While the 2/3 scaling was most closely observed in the homogeneous cluster, the outliers still exhibited a power law relationship between mean velocity and displacement of MEs that approached the theoretical value of 2/3. This indicates that the relationship between mean velocity and displacement may be a more basic principle of goal-directed voluntary movements than the shape of the MEs. We argue here that this relationship may be related to movement vigor. Movement vigor can vary among healthy individuals as some individuals move consistently faster than others. These between-subject differences in movement vigor have been observed in eye (Choi et al., 2014; Bargary et al., 2017; Reppert et al., 2018) and reaching movements (Reppert et al., 2018); and may be related to differences in how the CNS of each subject defines reward as a function of time (Choi et al., 2014). In fact, in a number of motor control models (Shadmehr et al., 2010, 2016; Rigoux and Guigon, 2012; Berret and Jean, 2016), it is thought that movement vigor is related to how the brain evaluates the usefulness of movements. In our proposed concept of MEs, while the 2/3 relationship observed between mean velocity and displacement seems to be conserved between and within subjects, the *K*-value that is observed within Equation (2) (see Supplementary material) shows between-subject variability (Supplementary Tables 1–3), especially for MEs in the homogeneous cluster. We know from Equation (2) (see Supplementary material) that *K* is related to mean velocity of MEs (i.e., for a given value of D, changing the value of *K* will necessarily alter the mean velocity). This may indicate the subject-specific representation of how the CNS evaluates the importance of the cost of time. Our results show that most subjects exhibit a different *K*-value, albeit only for MEs within the homogeneous cluster (Supplementary Table 3). This between-subject variability all but disappears for MEs within the outlier set. So, it is possible that the subject-specific movement vigor can be represented by the value of *K* within the homogeneous cluster because these movements are deemed already optimal by the CNS as they closely mirror the theoretical trajectory and thus, are likely performed in a feedforward manner. Conversely, the lack of variability in *K*-value within the outliers set (Supplementary Table 3) may be a steady-state level of movement vigor exhibited by the CNS while exploring the movement sub-space and relying on feedback. The higher *K*-values and consistency between subjects may be a byproduct or a necessary condition of the sensorimotor integration required for feedback control. This is supported by the fact that MEs in the homogeneous cluster had on average greater mean velocity, and shorter duration than MEs in the outliers set. Prior work has demonstrated that feedforward responses are significantly faster than feedback responses (Leonard et al., 2009; Tsao et al., 2009). Thus, the fact that the MEs in the homogeneous cluster are performed faster and are shorter is in line with the hypothesis that they are part of the feedforward system while the slower and longer MEs in the outliers cluster allow for feedback and sensory motor integration.

There are limitations to the current study. Several studies have suggested that invariances such as the 2/3 power law and planar invariances might arise from musculoskeletal mechanics rather than neural control (Sternad and Schaal, 1999; Gribble and Ostry, 2000). Others proposed alternative hypotheses to explain the control of movement such as the equilibrium-point hypothesis (Fel’dman, 1966) and the uncontrolled manifold hypothesis (Scholz and Schöner, 1999). Unfortunately, as only one anatomical landmark was tracked in this study, it is not possible to exclude the possibility that similar sources might explain the regularities of the MEs. However, while results from Miranda et al. (2018) suggest that MEs are generated within the CNS and are part of the motor planning process, future studies are required. Furthermore, a larger number of males were recruited than females (this was coincidental). While the sample size does not afford the possibility of comparing the results based on sex, prior work is inconclusive regarding differences in upper-limb movement patterns during various tasks (e.g., Gromeier et al., 2017; Nakatake et al., 2017). As such, further studies on the impact of sex on MEs are needed.

## 5 Conclusion

The current study confirms the results of Miranda et al. (2018) that 3D random movements can be decomposed into sub-units termed MEs within individual body axes. Furthermore, we demonstrate here that the variability in ME shape can be defined by a small number of patterns and that MEs that best display a bell-shaped curve are distributed unevenly across the different body axes.

## Data availability statement

The raw data supporting the conclusions of this article will be made available by the authors, without undue reservation.

## Ethics statement

The studies involving humans were approved by the University of Massachusetts Amherst Institutional Review Board. The studies were conducted in accordance with the local legislation and institutional requirements. The participants provided their written informed consent to participate in this study.

## Author contributions

J-FD: Conceptualization, Investigation, Supervision, Writing – review and editing. BO: Data curation, Formal analysis, Methodology, Writing – original draft. JM: Conceptualization, Writing – review and editing. SL: Conceptualization, Investigation, Supervision, Writing – review and editing.
