# Supplementary material for: Understanding voluntary human movement variability through data-driven segmentation and clustering
Source: Front Hum Neurosci. 2023 Nov 28;17:1278653. doi: 10.3389/fnhum.2023.1278653 (PMC10713770; doi:10.3389/fnhum.2023.1278653)
Supplement: Supplementary file 1 [file Data_Sheet_1.pdf]

Supplementary Materials

Description of movement elements (MEs)

The velocity profile of MEs is approximately bell-shaped and similar to that of 2D point-to-point reaching movements:

$$v(t) = D\left[\frac{30}{{t_f}^5}\, t^4 - \frac{60}{{t_f}^4}\, t^3 + \frac{30}{{t_f}^3}\, t^2\right] \tag{1}$$

where t_f is the duration of the movement, and D is the displacement. The mean velocity of movement $\bar{v}$ and its displacement D scale through a 2/3 power law according to the following equation:

$$\bar{v} = \frac{D^{2/3}}{60^{1/3}K^{1/6}} \tag{2}$$

such that $\bar{v} \propto D^{\alpha}$ with α being defined as the scaling exponent equal to 2/3 and where K is a constant. The average shape of the velocity profile of the MEs and the scaling between the mean velocity and displacement of MEs followed equations (1) and (2), respectively. However, variability around the cost function was observed.

Methods

Example of the movement patterns that subjects were asked to perform (Fig.S1).

Example of the movement patterns performed by 2 subjects during the two repetitions of the random movement task. As can be seen in the Figure, the movement patterns differed between repetitions for each subject and between each subject. This is representative of the data obtained for each subject enrolled in the study.

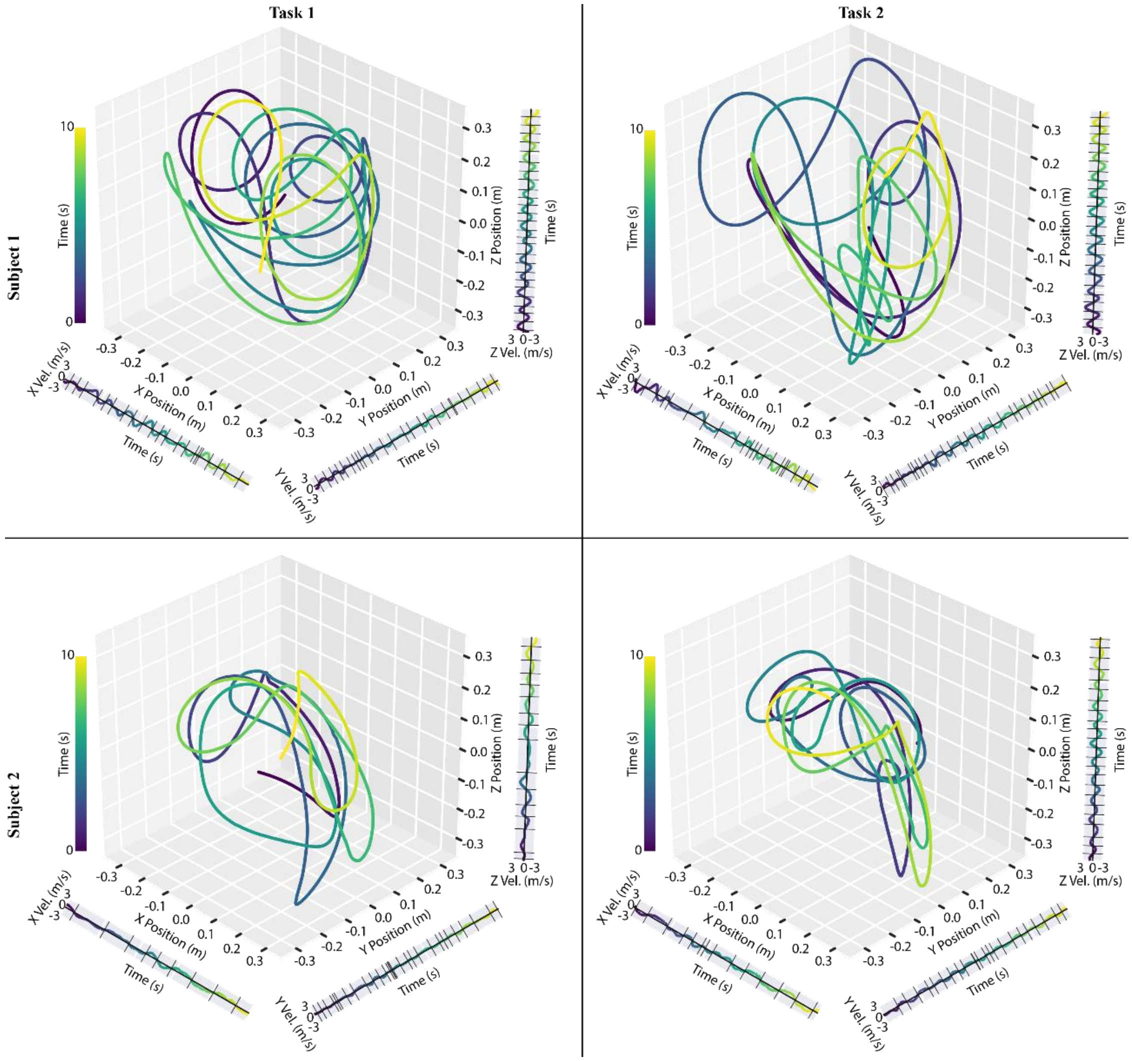

Figure S1 Example of a random movement (10s) performed by two of the subjects and its decomposition into movement elements within each of the three axes. Note that the pattern performed by both subjects differ and that the pattern performed during the different trials also differs within and between subjects.

ME extraction and removal of MEs below threshold

The velocity time-series, in each Cartesian axis (antero-posterior, medio-lateral, and rostro-caudal) (see Figure S2), was decomposed using zero crossings such that the continuous movement was segmented into MEs of zero initial and terminal velocities. Therefore, continuous 3D movement was viewed as a series of one-dimensional point-to-point reaching movements. The selection of the coordinate system was originally based on the best fit of the MEs to the

theoretical model proposed by Hoff (Hoff, 1994) (internal communication, data not published). We acknowledge that several other coordinate systems have been examined in the context of motor control, where there has been a focus on limb-specific coordinate systems or those aligned with specific body parts involved in balance and spatial orientation such as the semicircular canals or the extraocular muscles/eyes (Soechting & Flanders, 1992). This seminal work highlights that there is evidence for the use of several reference frames in the context of motor control. Furthermore, they describe evidence of different coordinate systems, such as Cartesian or spherical coordinate system. As such, while additional work linking MEs, and the coordinate system/reference frame used to extract them, to specific neurophysiological responses is needed, our current data suggests that using a Cartesian coordinate system originating at the center of mass is optimal.

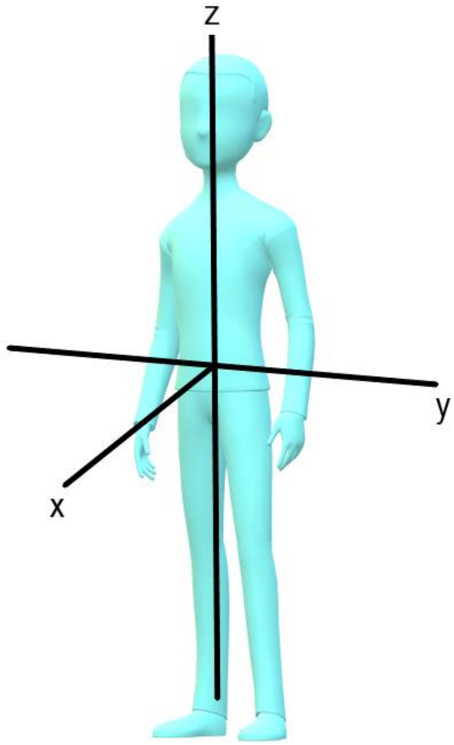

Figure S2. Visual representation of the coordinate system used in the current study where the x-axis represents the antero-posterior direction, the y-axis represents the medio-lateral direction, and the z-axis represents the vertical direction.

ME thresholds were then applied and each of the remaining ME was then spatially and temporally normalized to enable direct comparison. *Equation (1)* can be rewritten as:

$$v(\acute{t}) = \frac{D}{t_f}[30\acute{t}^4 - 60\acute{t}^3 + 30\acute{t}^2] \quad (3)$$

where $\acute{t} = t/t_f$, and D/t_f can be interpreted as the mean velocity. This equation demonstrates that MEs can be viewed as a template pattern that is stretched spatially by the mean velocity and

temporally by percent completion. Therefore, we normalized each ME by dividing it by its mean velocity and resampling it to 50 samples. Fifty samples were chosen because 500ms was close to the average duration of the extracted unnormalized MEs.

Dense cluster identification and definition of optimal parameters

The boundary between the dense and sparse region needed to be defined using an objective measure of cluster fitness to properly identify the region containing the MEs having a similar shape, similar to what we have previously done (Oubre et al., 2020). To that end, we needed to identify the adequate values for the two parameters that define density in the DBSCAN algorithm: γ and ε. When running DBSCAN on our dataset, a ME p would be considered part of a cluster only if there were at least γ other MEs within a sphere of radius ε centered around p. In our current implementation, the value of γ was kept constant at 5 as was previously suggested (Pedregosa et al., 2012). Therefore, to adequately define a dense region within our dataset, we needed to assess the impact of the value of ε (Fig.S3A). As we ran a search for the proper value of ε, we observed that as ε increases, both the number of MEs in the

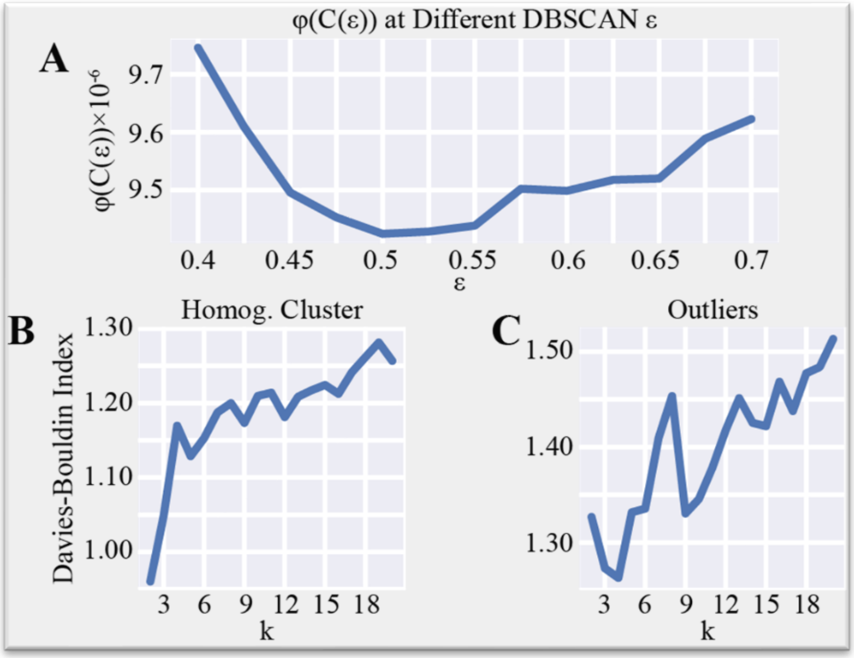

Figure S3: Illustration of the results for the search of different values for A) ε for the DBSCAN algorithm, B) k in the homogeneous cluster, and C) k in the outlier cluster.

identified homogenous cluster and the variance associated with discrepancies in the shape among the MEs of the identified homogenous cluster increased, but at different rates. Thus, there appeared to be a trade-off between the inclusiveness and variance of the homogeneous cluster as the value of ε changes.

We defined a fitness measure that constructs a homogeneous cluster containing as many MEs as possible while keeping the variance low based on the following equation:

$$\varphi(\mathbb{c}) = \frac{\overline{\sigma_{\mathbb{c}}}}{|\mathbb{c}|'}$$

where $\mathbb{c}$ is a subset of all MEs defined by the different values of ε (i.e., the homogeneous cluster), $\sigma_{\mathbb{c}}$ is a vector whose i^{th} element is the standard deviation of the ith element of every ME vector in $\mathbb{c}$, and $|\mathbb{c}|'$ represents the size of $\mathbb{c}$. Therefore, a low value of φ is associated with an increased number of MEs in the homogeneous cluster and a reduction of the variance within that cluster. The optimal cluster size was defined as:

$$\varepsilon^* = \text{argmin}_{\varepsilon}\left(\varphi\big(\mathbb{c}(\varepsilon)\big)\right)$$

Sub-cluster identification

For the identification of sub-clusters, we initially opted to run the *k*-Means algorithm on the movement elements included in the homogeneous cluster with *k* values ranging from 1 to 20 and computed the Davis-Bouldin Index to identify the optimal value of *k* (Fig.S3B). However, most

cluster validity metrics — including the Davies-Bouldin Index — attempt to promote cohesion within a cluster and separation between clusters. Since the homogenous cluster is already defined as a single, dense region, these metrics emphasize the *k* = 1 solution because any larger *k* will result in clusters with poor separation and similar cohesion. As such, the *k*-Means algorithm was implemented in the outliers set (a much sparser set of data) to determine its optimal value of *k* (Fig.S3C), which was then also used in the homogeneous cluster despite there not being any overt sub-clusters (see Fig.S4 for a visualization of the clusters).

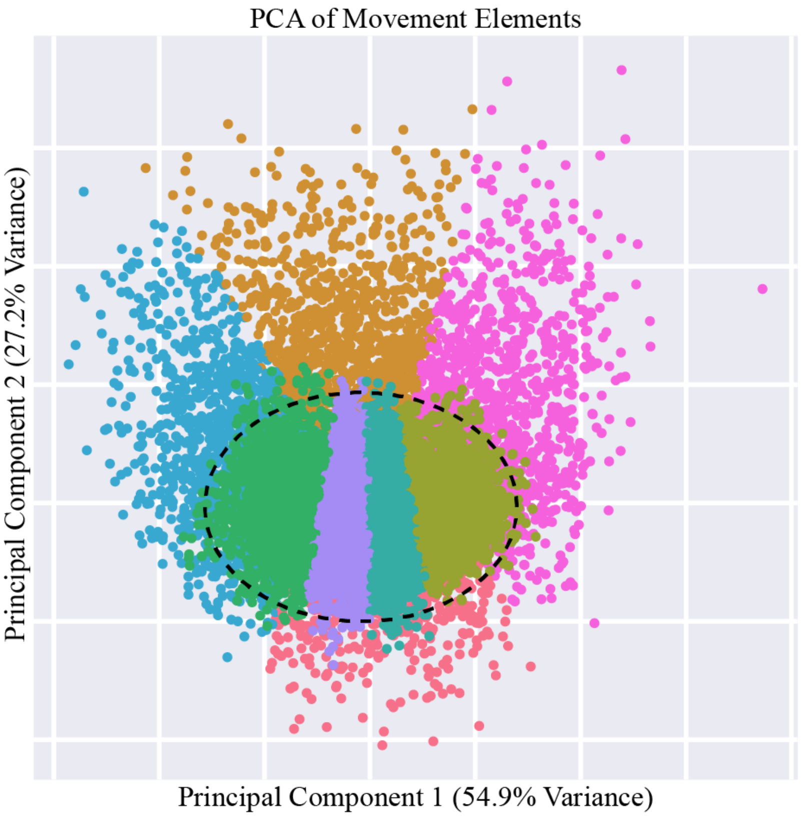

Figure S4: Visualization of the different cluster of the homogeneous set (within dashed ellipse) and outlier clusters.

Supplementary results

Figure S5 shows the average shape of the MEs from the homogeneous cluster at the subject level. These results emphasize the fact that in addition to closely matching the theoretical shape at the group level, ME shape is highly conserved across subjects in the homogeneous cluster. While not conclusive evidence, this further supports our assertion that MEs are an important aspect of motor control during voluntary movements.

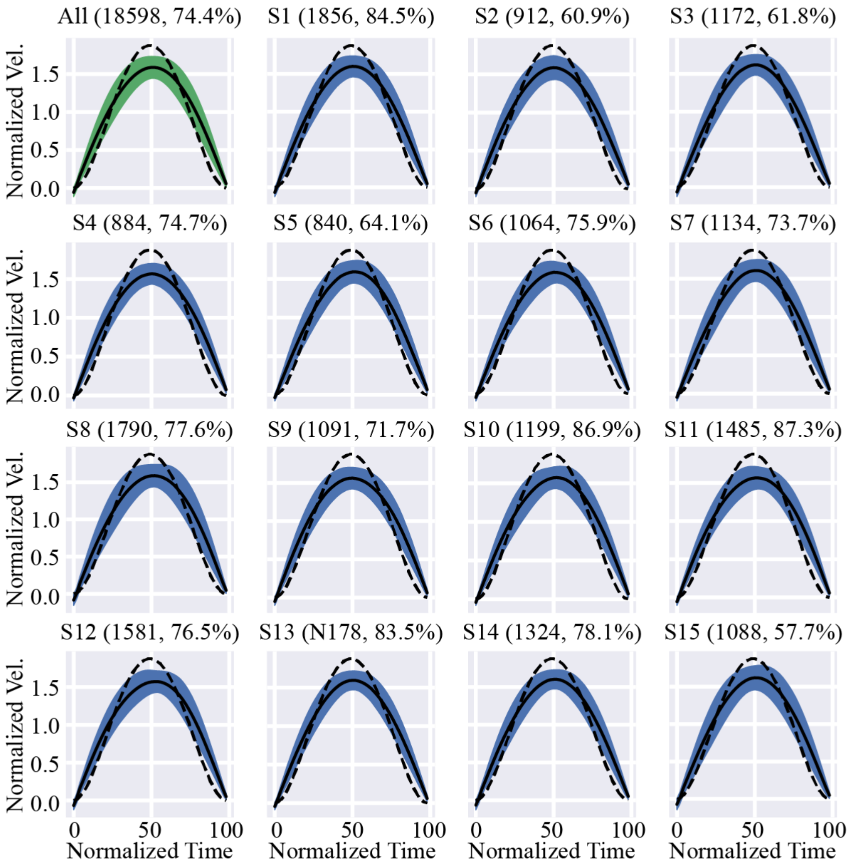

Figure S5: Illustration of the average movement element patterns from the homogeneous cluster across subjects. The dashed line represents the theoretical shape obtained from Equation 2. The black line represents the average shape of the data included in the graph. The shaded area represents the standard deviation (SD). The top left graph represents the aggregated data from all subjects as seen in Fig. 1a.

Since the length of the trials was relatively long, we wanted to confirm that this did not impact the results. As such, each trial was divided into two halves of equal length and the analysis was performed again. As can be seen in Fig. S6, while marginal differences could be observed in the shape of the MEs between the first and second halves, these did not have any impact on the overall results and interpretation of the results. We will acknowledge however, that the specific examination of the impact of fatigue on ME characteristics needs to be done in future studies.

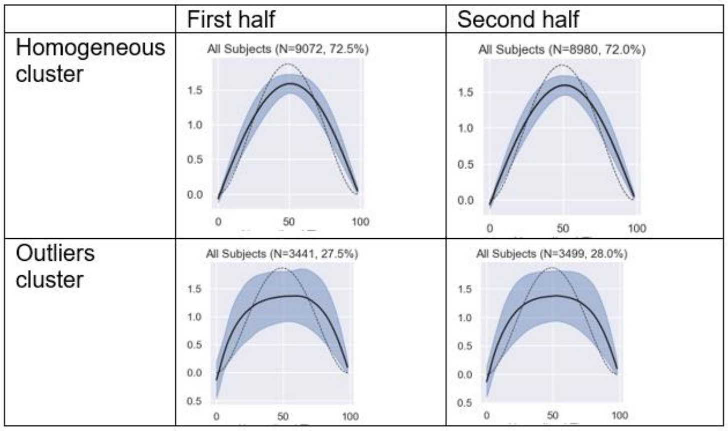

Figure S6: Illustration of the average movement element patterns from the homogeneous and outliers clusters when the trials were divided in half. The black line represents the average shape of the data included in the graph. The shaded area represents the standard deviation (SD).

In addition to these results, we examined the distribution of the MEs from the x, y, and z-axes as they relate to the different sub-clusters presented in Fig. 2A-B. First, it is important to note the disproportionate amount of z-axis MEs in the homogeneous set, and this is also representative of the distribution within the different sub-clusters (i.e., first (x-axis=20.73%, y-axis=32.51%, and z-axis=46.76%), second (x-axis=20.91%, y-axis=34.52%, and z-axis=44.57%), third (x-axis=18.51%, y-axis=36.65%, and z-axis=44.84%), and fourth (x-axis=32.08%, y-axis=32.12%, and z-axis=35.80%) sub-cluster identified in Fig.2A). Yet, within the homogeneous set, we identified that of the MEs extracted from the x-axis, 33.84%, 33.35%,13.59%, and 19.23% were part of the first, second, third, and fourth sub-clusters identified in Fig.2A, respectively. Similarly, we identified that of the MEs extracted from the y-axis, 34.40%, 35.69%, 17.44%, and 12.48% were part of the first, second, third, and fourth sub-clusters identified in Fig.2A, respectively. Finally, we identified that of the MEs extracted from the z-axis, 37.83%, 35.23%, 16.31%, and 10.63% were part of the first, second, third, and fourth sub-clusters identified in Fig.2A, respectively. This is expected as, by design, the MEs within the homogeneous set closely match the theoretical model. Within the outliers set, the overall distribution within and between sub-clusters differs such that the distribution in the first (x-axis=30.57%, y-axis=57.44%, and z-axis=12.00%), second (x-axis=34.53%, y-

axis=50.41%, and z-axis=15.06%), third (x-axis=39.10%, y-axis=47.51%, and z-axis=13.39%), fourth (x-axis=45.35%, y-axis=42.10%, and z-axis=12.55%) sub-cluster identified in Fig.2B differs from the one observed in the homogeneous set. This can also be observed from the MEs extracted from the x-axis as 35.91%, 20.24%, 22.43%, and 21.42% were part of the first, second, third, and fourth sub-clusters identified in Fig.2B, respectively. Similarly, we identified that of the MEs extracted from the y-axis, 46.80%, 20.49%, 18.91%, and 13.79% were part of the first, second, third, and fourth sub-clusters identified in Fig.2B, respectively. Finally, we identified that of the MEs extracted from the z-axis, 38.58%, 24.16%, 21.03%, and 16.23% were part of the first, second, third, and fourth sub-clusters identified in Fig.2B, respectively. Overall, this indicates that MEs in the x-axis seem to deviate most from the theoretical model.

Table S1 highlights the mean values of alpha and K, and their respective standard deviation (SD), for each subject based on the MEs of the homogeneous and outlier clusters. In addition, overall mean±SD of each metric is provided. Statistical tests revealed significant differences between clusters, as described in the manuscript.

Table S2 shows the subject-by-subject comparison of the K values obtained from the MEs in the homogeneous cluster. Specifically, a one-way analysis of variance (ANOVA) was conducted to identify if each subject's K value for the MEs included in the homogeneous cluster were different. As stated in the manuscript, the ANOVA identified significant differences. To determine which comparisons were different, Tukey's posthoc test was run. The q values obtained from the test are reported in Table S2. Comparisons where Tukey's test identified a statistically significant difference between the mean K value of the MEs within the homogeneous cluster between 2 specific subjects are highlighted in grey. For instance, the mean K value of the

MEs within the homogeneous cluster of Subject 1 is significantly different from the mean K value of the MEs within the homogeneous cluster of Subjects 2-7, 9, and 15.

Table S1

Subject	Hom. Alpha	Hom. K Mean	Hom. K Std.	Outlier Alpha	Outlier K Mean	Outlier K Std.
1	0.691986	3.02E-05	5.76E-05	0.604314	0.000776	0.001401
2	0.640238	0.000331	0.000699	0.590824	0.008	0.023717
3	0.644982	0.000167	0.000424	0.591057	0.005235	0.023008
4	0.636992	0.000571	0.000945	0.582158	0.01361	0.040813
5	0.657094	0.000746	0.001037	0.583453	0.043234	0.411728
6	0.645862	0.000619	0.001522	0.566028	0.023921	0.099319
7	0.64572	0.000245	0.000441	0.605358	0.007589	0.027147
8	0.638547	8.09E-06	1.86E-05	0.594097	0.000118	0.0003
9	0.652275	0.000215	0.000344	0.582362	0.007229	0.0218
10	0.636668	6.91E-05	0.000103	0.609386	0.001072	0.00276
11	0.670838	5.50E-05	0.000111	0.617234	0.00059	0.001097
12	0.675786	2.45E-05	4.02E-05	0.645826	0.000508	0.00184
13	0.679443	5.78E-05	0.00015	0.603625	0.000893	0.002948
14	0.638705	6.35E-05	0.000191	0.566676	0.003239	0.016829
15	0.636671	0.000235	0.000457	0.588685	0.00869	0.026488
**All**	**0.702818**	**0.000129**	**0.000453**	**0.621265**	**0.004888**	**0.033242**

Table S 1 Subject-specific values of alpha and K from Equation 2 for the movements that were included in the homogeneous cluster and in the outlier cluster.

Table S2

Subjects	1	2	3	4	5	6	7	8	9	10	11	12	13	14
2	19.3													
3	9.5	9.6												
4	34.4	13.2	23.6											
5	44.7	22.6	33.3	9.44										
6	39.8	16.6	27.8	2.8	7.1									
7	14.8	5.0	4.9	18.9	28.6	22.8								
8	1.7	20.6	11.0	35.6	45.8	41.0	16.2							
9	12.6	6.7	3.0	20.4	30.0	24.4	1.8	14.0						
10	2.7	15.5	6.2	29.4	39.1	33.9	11.0	4.2	9.1					
11	1.8	17.0	7.4	31.6	41.6	36.5	12.5	3.5	10.4	0.9				
12	0.4	19.1	9.6	33.8	43.9	39.0	14.7	1.2	12.6	3.0	2.2			
13	1.9	16.1	6.9	30.0	39.6	34.5	11.7	3.4	9.7	0.7	0.2	2.2		
14	2.4	16.1	6.7	30.3	40.2	35.1	11.7	4.0	9.6	0.4	0.6	2.7	0.4	
15	14.0	5.5	4.2	19.2	28.9	23.1	0.6	15.4	1.2	10.3	11.8	13.9	11.0	10.9

Subjects

Table S 2 Comparison of subject-specific values of K from Equation (2) between subjects for the movements that were included in the homogeneous cluster. Numbers in the cells are the rounded q values for the specific comparisons. Shaded cells indicate a significant difference between the K value of the movement elements of those subjects that were included in the homogeneous cluster ($p < 0.05$).

Table S3 shows the subject-by-subject comparison of the K values obtained from the MEs in the outlier cluster. Specifically, a one-way ANOVA was conducted to identify if each subject's K value for the MEs included in the outlier cluster were different. As stated in the manuscript, the ANOVA identified significant differences. To determine which comparisons were different, Tukey's posthoc test was run. The q values obtained from the test are reported in Table S3.

Comparisons where Tukey's test identified a statistically significant difference between the mean K value of the MEs within the outlier cluster between 2 specific subjects are highlighted in grey. For instance, the mean K value of the MEs within the outlier cluster of Subject 1 is only significantly different from the mean K value of the MEs within the outlier cluster of Subject 5.

Table S3

Subjects	1	2	3	4	5	6	7	8	9	10	11	12	13	14
**2**	1.3													
**3**	0.8	0.6												
**4**	2.0	1.0	1.5											
**5**	7.3	7.0	7.8	5.0										
**6**	3.7	2.8	3.5	1.6	3.3									
**7**	1.1	0.1	0.5	1.0	6.4	2.7								
**8**	0.1	1.6	1.1	2.3	8.3	4.2	1.4							
**9**	1.1	0.1	0.4	1.0	6.6	2.8	0.1	1.3						
**10**	0.04	1.0	0.6	1.6	5.9	3.0	0.9	0.1	0.8					
**11**	0.03	1.1	0.7	1.8	6.3	3.3	1.0	0.1	1.0	0.1				
**12**	0.05	1.5	1.0	2.2	8.1	4.0	1.3	0.1	1.2	0.1	0.1			
**13**	0.02	1.1	0.7	1.8	6.4	3.3	1.0	0.1	0.9	0.02	0.04	0.1		
**14**	0.4	0.9	0.4	1.6	7.0	3.4	0.7	0.6	0.7	0.3	0.4	0.5	0.3	
**15**	1.5	0.2	0.8	0.9	7.3	2.9	0.2	1.9	0.3	1.1	1.3	1.7	1.3	1.1

Table S 3 Comparison of subject-specific values of K from Equation 2 between subjects for the movements that were included in the outlier cluster. Numbers in the cells are the rounded q values for the specific comparisons. Shaded cells indicate a significant difference between the K value of the movement elements of those subjects that were included in the outlier cluster ($p<0.05$).

References

Hoff, B. (1994). A model of duration in normal and perturbed reaching movement. *Biological Cybernetics*, *71*(6), 481–488. https://doi.org/10.1007/BF00198466

Miranda, J. G. V., Daneault, J.-F., Vergara-Diaz, G., Souza de Olieveira eTorres, A., Quixada, A., de Lemos Fonseca, M., … Bonato, P. (2018). Complex Upper-Limb Movements Are Generated by Combining Motor Primitives that Scale with the Movement Size. *Scientific Reports, In Press*. https://doi.org/10.1038/s41598-018-29470-y

Oubre, B., Daneault, J. F. J.-F. J., Jung, H. T. H. H.-T., Whritenour, K., Miranda, J. J. G. V. J. J. G. V., Park, J., … Lee, S. I. S. S. I. (2020). Estimating Upper-Limb Impairment Level in Stroke Survivors using Wearable Inertial Sensors and a Minimally-Burdensome Motor Task. *IEEE Trans Neural Syst Rehabil Eng*, *Epub*(3), 601–611. https://doi.org/10.1109/TNSRE.2020.2966950

Pedregosa, F., Varoquaux, G., Gramfort, A., Michel, V., Thirion, B., Grisel, O., … Duchesnay, É. (2012). Scikit-learn: Machine Learning in Python. *Journal of Machine Learning Research*, *12*, 2825–2830. https://doi.org/10.1007/s13398-014-0173-7.2

Soechting, J. F., & Flanders, M. (1992). Moving in three-dimensional space: frames of reference, vectors, and coordinate systems. *Annual Review of Neuroscience*, *15*, 167–191. https://doi.org/10.1146/ANNUREV.NE.15.030192.001123
